# Postpartum Ankle Pain: A Suspected Complex Regional Pain Syndrome or a Rare Cause of Septic Arthritis?

**DOI:** 10.7759/cureus.19509

**Published:** 2021-11-12

**Authors:** José Bernardo Ferreira, Nuno Maia, João Pedro Caetano, Diana Fernandes, Marília Rodrigues, Simão Serrano

**Affiliations:** 1 Physical Medicine and Rehabilitation, Centro Hospitalar de Leiria, Leiria, PRT; 2 Orthopaedics, Centro Hospitalar de Leiria, Leiria, PRT; 3 Internal Medicine, Centro Hospitalar de Leiria, Leiria, PRT; 4 Rheumatology, Centro Hospitalar de Leiria, Leiria, PRT

**Keywords:** immunity, arthritis, ankle tuberculosis, pregnancy, tuberculosis

## Abstract

Ankle tuberculosis is a relatively rare condition and may develop after hematogenous dissemination from the pulmonary origin, particularly in cases of immunosuppression. Both pregnancy and delivery are relatively immunosuppressive states, and immune modulations during these periods can contribute to the pathogenesis of disseminated tuberculosis. A 26-year-old mother presented with severe, continuous, and debilitating pain in the left ankle, lasting for three months after delivery and associated with fever. Inspection demonstrated ankle swelling and redness, with a cold and cyanotic forefoot. Ankle radiograph and musculoskeletal ultrasound evaluation were obtained. Tibiotalar joint arthrocentesis revealed purulent liquid suggestive of septic arthritis and an emergent arthroscopic washout of the ankle was performed. The synovial mycobacterial culture was posteriorly positive and the diagnosis established was both pulmonary and osteoarticular tuberculosis. A comprehensive rehabilitation program was then implemented to achieve maximum functional gains. This report presents a rare case of ankle tuberculosis diagnosed in the postpartum period. Early evaluation, treatment, and adequate rehabilitation interventions can be crucial to promote functionality and enhance the quality of life.

## Introduction

Tuberculosis (TB), an infectious disease caused by *Mycobacterium tuberculosis*, constitutes a major health problem [1] despite its global declining incidence, with 10 million new cases reported worldwide in 2019 [2]. While TB infects the respiratory system in 90% of cases, in 10%, it is extra-pulmonary and can infect the skeletal, nervous, lymphatic, and genitourinary systems. The foot and ankle are affected in only 0.1-0.3% of extra-pulmonary tuberculosis (EPTB) cases and only 1-4% of all osteoarticular TB cases [3]. The talus and calcaneus are the most affected foot bones [4].

TB emerges after inhalation of contaminated aerosol droplets, as mycobacteria reach the alveoli to be phagocytosed by macrophages. In cases of immunosuppression, granuloma formation is incomplete, allowing bacteria to spread contiguously to adjacent tissue. When liquefying granuloma erodes into a blood vessel, bacillus can spread by hematogenous dissemination and cause EPTB [3].

Pregnancy is a clinical condition characterized by a relatively immunosuppressive state to avoid maternal anti-fetus rejection. However, after delivery, cellular immune reconstitution leads to postpartum exacerbation of otherwise quiescent or latent infections. Those may contribute to the pathogenesis of disseminated tuberculosis, as the risk of developing tuberculosis is two-fold higher in early postpartum women [5].

We present a rare case of ankle tuberculosis in the postpartum period. This case highlights the importance of establishing an early diagnosis in this specific population, in whom immune modulations can lead to atypical presentations.

## Case presentation

A 26-year-old Caucasian woman was referred to the outpatient clinic of our Department of Physical Medicine & Rehabilitation (PM&R) for further evaluation of severe pain in her left foot, associated with local bluish skin discoloration and complaints of a colder extremity. Of note in her past medical history, she reported working as a factory employee and being a regular user of tobacco and recreational cannabinoids. The onset of symptoms occurred one month after giving birth to a premature baby (first gestation, 29 weeks, normal vaginal delivery) and seven months prior to the evaluation at our Department of PM&R. The first symptom reported by the patient was diffuse swelling located at the lateral side of the left ankle, with progressive worsening. She then developed pain and redness at the same location for the following three months and was treated with naproxen, with initial pain relief and reduced swelling. Two months later, despite anti-inflammatory treatment, symptoms aggravated, and foot pain became progressively debilitating, with cyanotic cold extremity. Within the succeeding two weeks, the patient presented with fever (38ºC) and deteriorated loading capacity, requiring the use of two crutches to walk.

**Figure 1:**
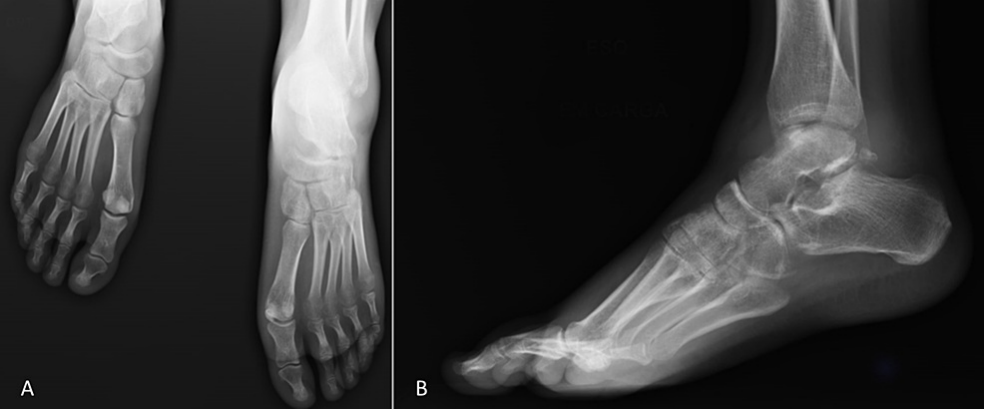
Weight-bearing ankle and foot radiography showed apparent joint effusion with articular defects (arthritis non-specific signs). A: Anteroposterior view. B: Left lateral view.

Upon inspection, there was diffuse swelling, redness, and low local temperature of the left ankle and foot. The forefoot was cold with anterior plantar cyanosis from the hallux to the third finger. Dorsalis pedis pulse was weak but apparently symmetrical. Ankle radiograph showed apparent joint effusion with articular defects (Figure 1).

**Figure 2:**
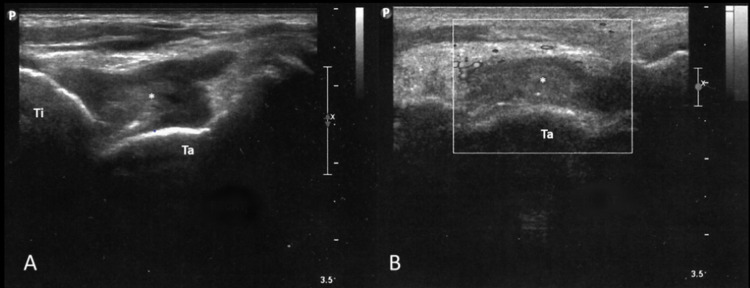
Ultrasound evaluation of the left ankle revealed moderate intra-articular hypoechoic effusion with heterogeneous content suggesting fluid with debris. A: Longitudinal plane of the tibiotalar joint. B: Transverse plane of the tibiotalar joint. * - tibiotalar joint effusion; P - proximal; Ti - tibia; Ta - talus.

A musculoskeletal ultrasound evaluation of the left ankle was performed as part of the physical examination and revealed moderate intra-articular hypoechoic effusion with heterogeneous content suggesting fluid with debris (Figure 2).

Following ultrasound evaluation of the ankle, an ultrasound-guided tibiotalar joint arthrocentesis was performed, with leakage of 8cc of purulent fluid suggestive of septic arthritis, which was then sent to cytological, bacterial, and mycobacterial analysis.

**Figure 3:**
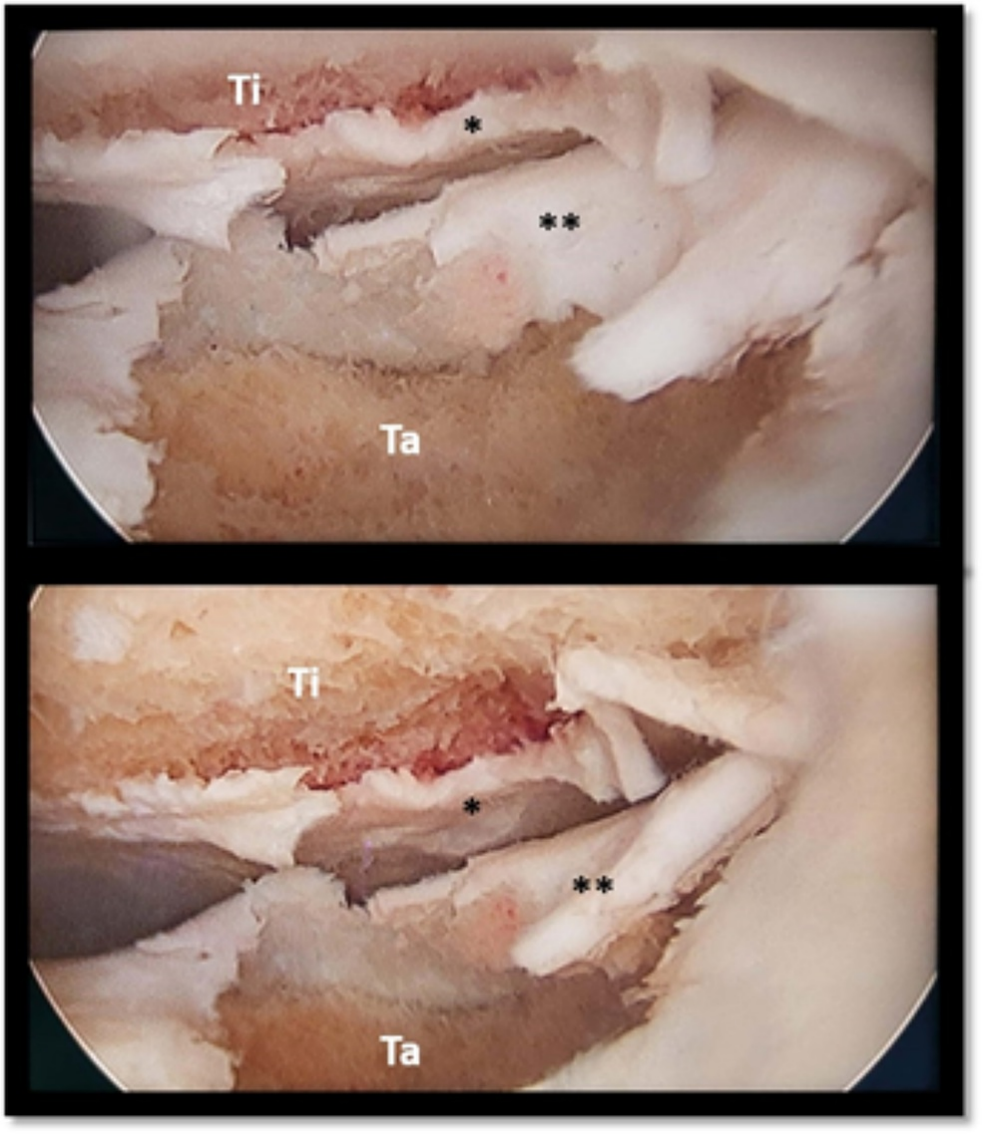
Arthroscopy of the left ankle detected a large chondral defect affecting the talus bone. * - tibial articular cartilage; ** - talar articular cartilage; Ti - tibia; Ta - talus.

The patient was then referred to our Orthopaedics Emergency Department to be submitted to an emergent arthroscopic washout of the ankle. During the procedure, a large chondral defect affecting the talus bone was detected (Figure 3). Therefore, an arthroscopic lavage and chondral regularization of the talus were performed. After surgery, she was admitted to the inpatient ward of our Department of Orthopaedics and started empirical vancomycin and rest associated with ice locally and elevation of the foot. At this time, the patient was able to walk, but with complete load restriction of the affected limb.

Laboratory test results are presented in Table 1. The synovial bacterial and mycological exams were negative as well as the blood cultures.

**Table 1:** Laboratory test results. ^a^ Reference range refers to the values used at the Leiria Hospital Center laboratory.

Variable	Result	Reference range ^a^
Blood count		
Hemoglobin (g/dl)	12.7	11.5-16.0
Hematocrit (%)	38.6	35.0-47.0
Platelet count (per μl)	641,000	150,000-500,000
White blood cells count (per μl)	10,600	4,000-10,000
Differential white blood cells count (per μl)		
Neutrophils	9,300	1,800-8,000
Lymphocytes	1,000	1,500-6,500
Monocytes	300	0-800
Eosinophils	0	0-600
Basophils	0	0-200
Acute phase reactants		
Erythrocyte sedimentation rate (mm/hour)	49	<15
High sensitivity C-reactive protein (mg/l)	120.3	<5.0
Synovial chemistry analysis		
Glucose (mmol/l)	0.5	3.9-6.0
Protein (g/l)	63.8	10.0-30.0
pH	8.0	-
Synovial cytology analysis		
Color/aspect	Purulent	-
Polymorphonuclear leukocytes (%)	96	-
Mononuclear leukocytes (%)	1	-
Non-leukocyte cells (%)	3	-

During the hospital stay, fever persisted for a few days and the patient noticed a cough with yellow to green sputum. Chest radiography revealed opacity on the left lung apex, suggestive of tuberculosis. Both the tracheobronchial aspirate and the bronchoalveolar lavage of the left superior lobe were positive for mycobacteria, with frequent alcohol-acid resistant bacilli. For this reason, the patient started medical treatment with tuberculostatics. Meanwhile, the synovial culture was positive for mycobacterium. Therefore, the patient was diagnosed with both pulmonary and osteoarticular tuberculosis.

After discharge, the patient was seen at the outpatient clinic of the Department of PM&R to reassess clinical condition, prescribe medication for pain control (tapentadol 50 mg orally twice a day and metamizole 575 mg orally pro re nata [prn]), and delineate a daily rehabilitation program at the hospital outpatient clinic, consisting of soft and progressive ankle active-assisted range of motion exercises and analytic muscular strengthening of ankle muscles (initially isometrics due to pain). Progressive weight-bearing gait training was also introduced to achieve the best possible function in activities of daily living and to evaluate pain response during gait.

One year after diagnosis, the patient was re-evaluated and could perform gait with only one crutch, presenting with mild pain during weight-bearing (six out of 10 on the pain visual analog scale) and almost no pain at rest, except for occasional episodes of night pain. There was also improvement in ankle joint mobility. The patient was then prescribed a carbon fiber ankle-foot orthosis to reduce pain during gait by facilitating support and propulsion of the affected extremity during the stance phase. Six months later, at a new consultation, she was able to perform gait without any walking aid, still with moderate pain during weight-bearing (six out of 10 on the pain visual analog scale).

## Discussion

This case underlines the importance of prompt identification and diagnosis of ankle tuberculosis, especially in the postpartum period. As noted in this clinical case, diagnosing this condition can be challenging, considering its atypical presentation and lack of specificity in diagnosis.

Traditionally, the main manifestation is chronic synovitis, but some patients can present with swelling in the form of a “cold abscess,” without the classic signs of acute inflammation [6]. These are associated with slow-growing infections and may mimic other diseases [3]. Furthermore, classical systemic TB symptoms are frequently absent and test results (such as inflammatory markers) may not be specific. The definitive diagnosis implies the demonstration of bacteria with special staining techniques (Ziehl-Neelsen) or isolation in culture ideally from biopsy specimens [1]. Hence, a diagnostic delay is quite common, with a reported range of one month to 15 years [3].

The primary treatment objectives for osteoarticular TB include controlling the infection, limiting deformity, maintaining mobility, and reducing discomfort. Medical treatment with tuberculostatic agents represents the mainstay of foot and ankle tuberculosis therapy. It should be administered as early as possible for at least six months but can be extended for up to 24 months in resistant cases, constituting a safe option during lactation [3]. All breast-feeding patients treated with isoniazid should also be prescribed vitamin B6 (pyridoxine) [7]. Surgical treatment should always be regarded as an adjuvant to medical treatment. The main goals of surgery are the removal of dead tissue and neovascularization of salvageable tissue [8]. It constitutes a viable option not only for cases resistant to tuberculostatic agents but also late-disease presentation cases with either abscess formation or osteochondral destruction and deformity due to soft tissue necrosis. Arthroscopy constitutes a valuable tool, as it identifies early-stage ankle tuberculosis macroscopic lesions and assists in local disease control, with debridement [6]. Arthrodesis is reserved for advanced cases with collapsed or narrowed joint space, aiming to reduce/neutralize pain due to secondary osteoarthritis, as the last line of treatment [3].

From our literature review, we did not identify previous reports of ankle TB diagnosed in the postpartum period. The reported cases in the literature of osteoarticular TB whether in pregnancy or postpartum period address spinal TB (Pott’s disease), psoas abscess, sacroiliitis, and radius involvement.

This clinical case is characterized by some poor prognostic factors. First, the postpartum period is associated with worse TB outcomes, as there may be a paradoxical treatment response [5]. Furthermore, the diagnostic and treatment delay can lead to increased clinical severity at presentation and a higher risk of arthrosis and deformities [9]. Also, medical treatment is considered most effective at early disease stages limiting disability and promoting healing [4]. The atypical clinical presentation verified on this patient, with low local temperature and diffuse swelling of the left foot and ankle, may explain the initial referral delay to a specialized consultation. In addition to that, a biopsy of the lesion was not performed at the time of surgery, a fact that could have expedited the diagnostic process. Nevertheless, even though adverse prognosis and marked functional limitations characterized this case, the effectiveness of the implemented rehabilitation plan was fundamental for the patient to reacquire the capacity of walking as functionally as possible, limiting pain. In addition, the prescribed orthotic device enabled the patient to achieve more quickly the primary objectives established by the physiatrist.

## Conclusions

When a patient presents with atypical signs of septic arthritis of the ankle with long-term evolution, particularly in the postpartum period, osteoarticular TB should be considered. Early evaluation and treatment can be decisive prognostic factors.

When the prognosis is adverse, a comprehensive rehabilitation plan with realistic goals and including the prescription of useful orthotic devices is of utmost importance. Those aspects can maximize patient functionality and enhance the quality of life.
